# Cluster of SARS-CoV-2 Gamma Variant Infections, Parintins, Brazil, March 2021

**DOI:** 10.3201/eid2801.211817

**Published:** 2022-01

**Authors:** Juliana F. da Silva, Roberto J. Esteves, Charlene Siza, Elaine P. Soares, Tatyana C. Ramos, Evelyn C. Campelo, Cristiano F. da Costa, Leila C. de Alencar, Rafaela P. Cavalcante, Clerton R. Florêncio, Tirza P. Mattos, Maria G. Bonecini-Almeida, Luciana Silva-Flannery, Barbara J. Marston, Juliette Morgan, Mateusz Plucinski, Felipe Naveca, CDC Brazil Investigation Team

**Affiliations:** US Centers for Disease Control, Atlanta, Georgia, USA (J.F. da Silva, C. Siza, L. Silva-Flannery, B.J. Marston, M. Plucinski);; US Centers for Disease Control and Prevention, Brasilia, Brazil (R.J. Esteves, J. Morgan);; Secretaria Municipal de Saúde de Parintins, Parintins, Brazil (E.P. Soares, R.P. Cavalcante, C.R. Florêncio);; Fundação de Vigilância em Saúde do Amazonas, Manaus, Brazil (T.C. Ramos, E.C. Campelo, C.F. da Costa, L.C. de Alencar);; Laboratório Central de Saúde Pública do Amazonas, Manaus (T.P. Mattos);; Fundação Oswaldo Cruz, Rio de Janeiro, Brazil (M.G. Bonecini-Almeida);; Instituto Leônidas and Maria Deane, Manaus (F. Naveca); 1 Team members are listed at the end of the article.

**Keywords:** COVID-19, 2019 novel coronavirus disease, coronavirus disease, severe acute respiratory syndrome coronavirus 2, SARS-CoV-2, viruses, respiratory infections, zoonoses, Gamma variant, P.1 variant, reinfection, Brazil

## Abstract

High case counts after the Gamma (P. 1) variant of severe acute respiratory syndrome coronavirus 2 emerged in Brazil raised concerns that previously infected persons might become reinfected. Investigation of a cluster of coronavirus disease cases in Parintins, in the Brazilian Amazon, suggested household transmission but did not identify high rates of reinfection.

In Parintins, Brazil, an increased rate of coronavirus disease (COVID-19)–associated hospitalization, from 75.5 cases/100,000 persons in November 2020 to 397 cases/100,000 persons in February 2021, led to an unprecedent health crisis on this island. The outbreak coincided with emergence of the Gamma (P.1) variant of severe acute respiratory syndrome coronavirus 2 (SARS-CoV-2), raising concern that the variant was causing infection even in persons who previously had COVID-19 (*1*). In March 2021, the Municipal Health Department of Parintins, in collaboration with the US Centers for Disease Control and Prevention (CDC), investigated recently infected persons and their household contacts to identify circulating SARS-CoV-2 variants, assess epidemiologic and laboratory evidence of previous SARS-CoV-2 infection in infected persons, and assess intrahousehold transmission.

We used the COVID-19 surveillance database in Parintins to identify persons >18 years of age who had a positive SARS-CoV-2 antigen test result (Panbio COVID-19; Abbott, https://www.abbott.com) in the previous 3 days. On March 4 and 5, 2021, the 22 case-patients identified were visited at home, and all adults able to provide written consent were invited to participate; 90 persons (22 index patients, 68 household contacts) agreed. An index case-patient was defined as the person with the earliest symptom onset date in the household; for all but 1 household, index case-patients were the same persons initially identified in the surveillance database. All participants responded to a questionnaire and provided nasopharyngeal swab and dried blood spot samples; nasal swab samples for antigen testing (BINAXNow; Abbott) were obtained from household contacts only.

We tested nasopharyngeal swabs by reverse transcription PCR (RT-PCR) (Allplex 2019-nCov Assay; Seegene, https//www.seegene.com) and by a variant-of-concern–specific RT–PCR protocol (*2*). Samples with cycle threshold (C_t_) values <30 underwent whole-genome sequencing (COVIDSEQ; Illumina, https://www.illumina.com) (*1*) to confirm screening results. We tested dried blood spot samples for SARS-CoV-2 IgG (FlexImmArray; Tetracore, https://tetracore.com).

We analyzed data by using RStudio version 1.4.1106 (https://www.rstudio.com). We assessed differences in proportions and odds ratios by using Fisher exact tests. The study was conducted in accordance with applicable federal law and CDC policy and approved by local health authorities in Brazil.

At the time of the investigation, diagnosis in the region was based on clinical findings or SARS-CoV-2 point-of-care IgG test results. A previous COVID-19 diagnosis was defined as illness diagnosed by a physician as COVID-19 or history of fever and >1 other COVID-associated sign/symptom >3 months earlier.

Of 90 participants, results of SARS-CoV-2 antigen testing, RT-PCR, or both were positive for 54 (60%). Among 45 persons tested with variant-of-concern RT-PCR, whole-genome sequencing, or both, the Gamma variant was detected for 68.9%. We found no significant difference in the proportion of symptomatic infections caused by the Gamma variant (87.0%) and other variants (92.8%), (odds ratio [OR] 0.52, 95% CI 0.01–6.05; p = 0.56). When we excluded persons for whom the virus genotype differed from that of the household index case-patient, the proportion of infected household contacts was 45% (14/31) in households with a Gamma variant index case-patient and 25% (3/12) with a non–Gamma variant index case-patient (OR 2.42, 95% CI 0.47–16.59; p = 0.22).

Because all 14 non-gamma samples had C_t_ values >32, none was successfully sequenced; in contrast, 27/31 Gamma specimens were sequenced. Gamma variant sequences were substantially diverse; overall samples differed by <17 single-nucleotide polymorphisms. However, samples from persons in the same household largely clustered; of 21 samples from households with >1 sequence available, 17 (81%) differed by <2 nt (Figure).

**Figure:**
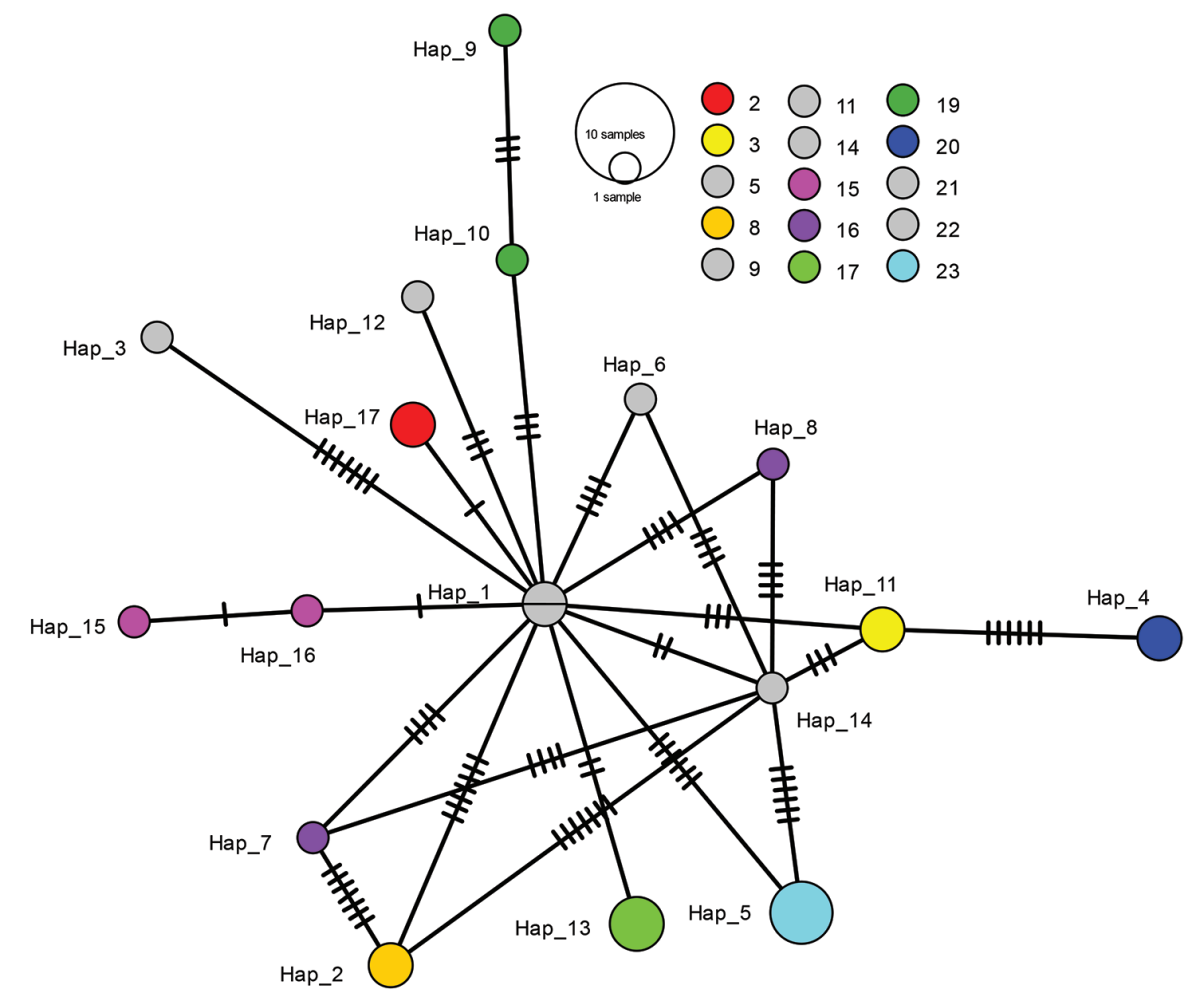
Network showing relationship between Gamma variant severe acute respiratory syndrome coronavirus 2 (SARS-CoV-2) sequences from household members involved in investigation of cluster of SARS-CoV-2 Gamma (P.1) variant infections, Parintins, Brazil, March 2021. Nodes represent unique sequences, and dashes connecting nodes denote the number of base-pair differences between sequences. Samples are from 27 household members from 15 households, and colors denote samples from the same household; gray indicates samples from households from which only 1 virus sequence was available. Node size is proportional to the number of samples with a given sequence. Hap, haplotype.

Among 31 patients with Gamma variant infection, none met our definition of having a previous diagnosis of COVID-19 (Table). In contrast, 3 (21.4%) of 14 persons with a non–Gamma variant infection and 16 (44.4%) of 36 persons tested by RT-PCR but found to be uninfected had evidence of previous infection or illness (p = 0.026 and p<0.0001, respectively).

**Table:** History of previous infection among 81 persons infected with SARS-CoV-2 Gamma variant, infected with SARS-CoV-2 non–Gamma variant, or not infected with SARS-CoV-2, Parintins, Brazil, March 2021*

Infection status	No. (%)			p value (Gamma . negative)‡
	Gamma variant, n = 31	Non–Gamma variant, n = 14	Negative, n = 36†	
Previous infection according to clinical history				
Yes	4 (13)	4 (29)	18 (50)	0.0016
No	27 (87)	10 (71)	18 (50)	
IgG result at time of investigation				
Positive	5 (16)	10 (71)	25 (69)	<0.0001
Negative	26 (84)	4 (29)	11 (31)	
Previous infection according to clinical history and positive IgG at time of investigation				
Yes	0	3 (21)	16 (44)	<0.0001
No	31 (100)	11 (79)	20 (56)	

*9/90 patients refused nasopharyngeal swab sampling and were not included in the analysis. SARS-CoV-2, severe acute respiratory syndrome coronavirus 2. †By PCR. ‡By Fisher exact test.

We determined that the Gamma variant was responsible for a high proportion of infections in this cluster; we did not find evidence of Gamma variant infection in persons with previous COVID-19. The clustering of sequences by household suggests intrahousehold transmission, which might reflect the difficulty of isolating case-patients at home, especially in large households.

Our findings are limited because the participants were not selected randomly and results may not be generalizable. For some participants, IgG could reflect response to current rather than previous infection, and the use of serologic and clinical criteria might overestimate the number of previous infections. In addition, sequencing success among Gamma and non–Gamma variants might be affected by different viral loads or times of infection relative to time of sampling.

According to the limited epidemiologic and laboratory data available, vaccines currently deployed in Brazil seem to protect against the Gamma variant (*3*,*4*). Since the investigation, Parintins has accelerated its immunization campaign, resulting in an ≈8-fold increase in the percentage of persons receiving the first vaccine dose during January–June 2021. Although Gamma variant infections did not increase among persons with a history of prior COVID-19, vaccination of all eligible persons and implementation of measures to mitigate intrahousehold transmission will help reduce the spread of SARS-CoV-2.

Appendix Additional results from study of cluster of SARS-CoV-2 Gamma variant infections, Parintins, Brazil, March 2021.
