# Supplementary material for: Cluster of SARS-CoV-2 Gamma Variant Infections, Parintins, Brazil, March 2021
Source: Emerg Infect Dis. 2022 Jan;28(1):262–4. doi: 10.3201/eid2801.211817 (PMC8714215; doi:10.3201/eid2801.211817)
Supplement: Appendix — Additional results from study of cluster of SARS-CoV-2 Gamma variant infections, Parintins, Brazil, March 2021. [file 21-1817-Techapp-s1.pdf]

Article DOI: https://doi.org/10.3201/eid2801.211817

Cluster of SARS-CoV-2 Gamma Variant Infections, Parintins, Brazil, March 2021

Appendix

Appendix Table. Demographic and clinical characteristics of index patients and contacts in a cluster investigation of SARS-CoV-2 gamma variant (N = 90) — Parintins, Brazil, March 2021

	No. (%)		
Characteristics	Index cases (n = 22)	Household contacts (n = 68)	Total (n = 90)
Age, median (range), y	41.0 (22–75)	34.5 (18–82)	37 (18–82)
Male	11 (50.0)	31 (45.6)	42 (46.7)
Race			
Mixed race	16 (72.7)	57 (83.8)	73 (81.1)
Black	1 (4.6)	4 (5.9)	5 (5.6)
Indigenous	–	1 (1.5)	1 (1.1)
White	1 (4.6)	3 (4.4)	4 (4.4)
Unknown	4 (18.2)	3 (4.4)	7 (7.8)
Healthcare worker	3 (13.6)	3 (4.4)	6 (6.7)
Educational level			
High school/technical training or less	11 (50.0)	45 (66.2)	56 (62.2)
Some college or above	11 (50.0)	23 (33.8)	34 (37.8)
Comorbidities*	6 (27.3)	15 (22.1)	21 (23.3)
Received at least first dose of vaccine†	4 (18.2)	8 (11.8)	12 (13.3)

*Any medical conditions for which the patient was prescribed regular medications.

†Astra-Zeneca or CoronaVac (Sinovac Biotech) vaccines were available in Parintins at the time of data collection. Both are administered as a 2-dose series.
